# HBx Inhibits CYP2E1 Gene Expression via Downregulating HNF4α in Human Hepatoma Cells

**DOI:** 10.1371/journal.pone.0107913

**Published:** 2014-09-19

**Authors:** Hongming Liu, Guiyu Lou, Chongyi Li, Xiaodong Wang, Arthur I. Cederbaum, Lixia Gan, Bin Xie

**Affiliations:** 1 Department of Hepatobiliary Surgery, Daping Hospital & Institute of Surgery Research, The Third Military Medical University, Chongqing, China; 2 Department of Biochemistry and Molecular Biology, The Third Military Medical University, Chongqing, China; 3 Department of Pharmacology and Systems Therapeutics, Icahn School of Medicine at Mount Sinai, New York, New York, United States of America; 4 Chongqing Biomean Technology Co., Ltd, Chongqing, China; Yonsei University, Republic of Korea

## Abstract

CYP2E1, one of the cytochrome P450 mixed-function oxidases located predominantly in liver, plays a key role in metabolism of xenobiotics including ethanol and procarcinogens. Recently, down-expression of CYP2E1 was found in hepatocellular carcinoma (HCC) with the majority to be chronic hepatitis B virus (HBV) carriers. In this study, we tested a hypothesis that HBx may inhibit CYP2E1 gene expression via hepatocyte nuclear factor 4α (HNF4α). By enforced HBx gene expression in cultured HepG2 cells, we determined the effect of HBx on CYP2E1 mRNA and protein expression. With a bioinformatics analysis, we found a consensus HNF-4α binding sequence located on −318 to −294 bp upstream of human CYP2E1 promoter. Using reporter gene assay and site-directed mutagenesis, we have shown that mutation of this site dramatically decreased CYP2E1 promoter activity. By silencing endogenous HNF-4α, we have further validated knockdown of HNF-4α significantly decreased CYP2E1expression. Ectopic overexpression of HBx in HepG2 cells inhibits HNF-4α expression, and HNF-4α levels were inversely correlated with viral proteins both in HBV-infected HepG2215 cells and as well as HBV positive HCC liver tissues. Moreover, the HBx-induced CYP2E1 reduction could be rescued by ectopic supplement of HNF4α protein expression. Furthermore, human hepatoma cells C34, which do not express CYP2E1, shows enhanced cell growth rate compared to E47, which constitutively expresses CYP2E1. In addition, the significantly altered liver proteins in CYP2E1 knockout mice were detected with proteomics analysis. Together, HBx inhibits human CYP2E1 gene expression via downregulating HNF4α which contributes to promotion of human hepatoma cell growth. The elucidation of a HBx-HNF4α-CYP2E1 pathway provides novel insight into the molecular mechanism underlining chronic HBV infection associated hepatocarcinogenesis.

## Introduction

Hepatocellular carcinoma (HCC) is the fifth most common cancer globally and the second leading cause of cancer death in China, a country with the largest HCC population in the world [1]–[3]. The risk factors for HCC have been well defined and included hepatitis virus infection, cirrhosis, and alcohol consumption. More than 50% of HCC patients are Hepatitis B Virus (HBV) carriers, and chronic HBV infection has been regarded as the major etiological factor of HCC [4]. However, the mechanism by which HBV contributes to the development of HCC remains incompletely understood. Increasing evidence suggests that the X protein encoded by HBV genome plays a critical pathogenic role in HCC development [5], [6].

Cytochrome P450 2E1 (CYP2E1), a member of the cytochrome P450 mixed-function oxidase system, plays an important role in the metabolism of xenobiotics including ethanol, acetone, drugs and procarcinogens [7], [8]. So far, at least 18 families of CYP genes and 43 subfamilies have been identified [8], which account for almost 75% of the total drug metabolism in humans. CYP2E1 is one of the most abundant isoforms among all P450s [9]. In human liver, CYP2E1 is constitutively expressed and is induced under a wide variety of physiological or pathophysiologic conditions such as alcohol consumption, fasting, obesity and diabetes [10], [11]. Regulation of CYP2E1 expression is complex and could occur at transcriptional, translational and post-translational levels [12]. Genetic polymorphism in the 5′- regulatory region of CYP2E1 has also been shown to alter its gene expression [13], [14]. Although CYP2E1 has been extensively studied for many years, lots of this research effort has been focused on its role in drug metabolism and alcoholic liver diseases. Therefore, our knowledge of CYP2E1 in HCC is limited. Recently, gene array studies revealed that CYP2E1 was significantly down-expressed in HCC liver tissue [15]. Using a chemical-induced rat HCC model, Man et al showed that CYP2E1 expression declined along with the initiation, promotion and progression of HCC [16]. In clinical specimens from 85 HCC patients, Ho et al. found that 70% of the tumor tissues showed lower expression of CYP2E1, and decreased CYP2E1 is associated with poor prognosis of HCC [17]. These studies suggest that down-regulation of CYP2E1 may play an important role in HCC tumorigenesis. However, the mechanism of CYP2E1downregulation in HCC has so far not been elucidated.

Hepatocyte nuclear factors (HNFs) are liver-enriched transcription factors controlling multiple liver-specific gene expression and maintaining hepatocyte differentiation. These proteins belong to the nuclear hormone receptor superfamily which currently consist HNF1, HNF3, C/EBP, HNF4, and HNF6 [18]. As a key member of the HNF4 family, HNF-4α is indispensable for the hepatic epithelium formation during embryonic development and for epithelial phenotype maintenance of hepatocytes in mature liver [19], [20]. Expression of HNF-4α has been found to be at lower levels in HCC tissues compared to para- or non-cancerous liver tissue [21], [22]. Loss of HNF-4α accelerates HCC progression [21], whereas introduction of HNF-4α dramatically blocked the development of HCC in rats subjected to diethylinitrosamine administration [23], [24]. Thus, HNF-4α has been implicated as a key tumor suppressor in HCC development. Recent studies showed that it also plays important roles in regulating the expression and replication of HBV by stimulating the transcription of HBV pregenomic RNA [25], [26]. Overexpression of HNF4α enables replication of the HBV genome even in nonhepatic cell lines [25]. A reduction in the expression of HNF4α in liver cells reduces HBV replication in primary human hepatocytes [27] and in transgenic mice [28]. Currently, it is not clear whether expression of CYP2E1 is regulated by HNF-4α. It is also not known whether HNF-4α expression can be regulated by HBV viral protein in hepatocytes.

In this study, a hypothesis was proposed that HBx may inhibit CYP2E1 gene expression via HNF-4α. We found that ectopic overexpression of HBx inhibits CYP2E1 gene expression via downregulating HNF4α in cultured HepG2 cells and promotes hepatoma cell growth. Furthermore, using proteomics analysis,the changed proteins in the liver tissue from CYP2E1 knockout mice were evaluated. As overexpression of CYP2E1 dramatically inhibits hepatoma cell growth, our observation implies that down-regulation of CYP2E1 during chronic HBV infection may promote hepatocarcinogenesis.

## Materials and Methods

### Patients and mice tissue samples

#### Ethical Statement

Informed written consent was obtained from the patients for the collection of liver specimens, and the study protocol was approved by the Medical Ethics Committee in Daping Hospital, the Third Military Medical University.

5 pairs of primary HCC tissues and their adjacent tissues, and 5 cases of non-hepatitis B virus infection liver tissues from liver hemangioma (used as normal liver controls) were obtained from surgical resection in the Department of Hepatobiliary Surgery of the Daping Hospital, the Third Military Medical University. Each tissue was frozen immediately after surgery and stored in liquid nitrogen for later extraction of RNA/DNA and protein. All tumors were independently confirmed as HCC on haematoxylin and eosin (HE) stained sections by two pathologists. 5 cases of HCC tissues and their adjacent tissues, and 5 cases of normal liver tissues were processed into paraffin slides and stained for immunohischemistry.

The mice received humane care, and experiments were carried out according to the criteria outlined in the Guide for the Care and Use of Laboratory Animals and with approval of the Mount Sinai Animal Care and Use Committee.

### Materials

Human hepatocarcinoma cell lines HepG2 were maintained by our laboratory. HepG2.2.15, a cell line with HBV DNA sequences chromosomally integrated into HepG2 cells and capable of consistently expressing all the HBV encoded proteins, was kindly provided by the Infection Diseases Center of Southwest Hospital in our university. E47 and C34 cells, two stably transfected HepG2 cell lines harboring CYP2E1 recombinant gene or its empty vector as a control, respectively, were kindly gifts from Dr. Arthur Cederbaum in Mount Sinai Medical Center. The recombinant adenoviruses of AdHNF4α and AdGFP (control) were gifts from Dr. Wei-Fen Xie in the Second Military Medical University. The pGL3- Basic plasmid and the transfection reagent Lipofectamine 2000TM were bought from Invitrogen (USA). pMD18-T vector was purchased from TaKaRa Biotechnology (Dalian Co.). The pCMV-2B–FLAG-X (HBx gene expression vector) was a gift from the Institute of Viral Hepatitis affiliated to Chongqing Medical University. Anti-HBx, -CYP2E1 and –HNF4α antibodies were purchased from Abcam (USA) for both western blotting and immunochemistry.

### Cells and cell culture

HepG2 cell lines were maintained in 10% fetal bovine serum (Life Technologies, Inc.) in DMEM (Life Technologies, Inc.) and grown at 37°C in a 5% CO_2_ incubator. Before the experiment, cells were trypsinized and inoculated at a density of 1×10^5^ cells in a 24-well plate or 4×10^5^ cells in a 6-well plate.

### Construction for pGL3-CYP2E1 promoter reporter plamids

Human CYP2E1 (Gene ID: 219567) promoter DNA was amplified by PCR using primers listed in Table 1. The 1.4 kilobase pairs of the CYP2E1 promoter DNA was ligated into the pMD18-T vector using T4 DNA ligase and then transformed into E. coli. The pGL3-Basic vector was used to construct the expression vectors by subcloning PCR-amplified DNA of the CYP2E1 promoter into the XhoI/HindIII site of the pGL3-Basic vector (pGL3-CYP2E1-P). Similarly, deletion constructs were synthesized, annealed, and cloned into the EcoRV site in the pMD18-T vector, and then subcloned into pGL3-Basic vector. The PCR products were validated by their size with electrophoresis, and confirmed by DNA sequencing. Ten of the human CYP2E1 promoter constructs harboring sequential deletion of about 100-bp fragments from the 5′-ends were made with the corresponsive primers shown in Table 1.

**Table 1:** Primers used for making CYP2E1 promoter luciferase reporter gene constructs.

Construct Name	Sense (5′→3′)	Antisense (5′→3′)
CYP2E1-P-Luc	CATTGTCAGTTCTCACCTC	GGACACCAGCAGGAGGAAG
CYP2E1-P1-Luc	CAATGACTTGCTTATGTGG	GGACACCAGCAGGAGGAAG
CYP2E1-P2-Luc	CCACAAGTGATTTGGCTGG	GGACACCAGCAGGAGGAAG
CYP2E1-P3-Luc	TGCCAGTTAGAAGACAGAATG	GGACACCAGCAGGAGGAAG
CYP2E1-P4-Luc	CATAGAAGGTGGAAGAGGG	GGACACCAGCAGGAGGAAG
CYP2E1-P5-Luc	CCGGGATCAACAAAGACAAG	GGACACCAGCAGGAGGAAG
CYP2E1-P6-Luc	CTACAGCCAGAATATATACC	GGACACCAGCAGGAGGAAG
CYP2E1-P7-Luc	CTGGGGGCTGCTCAGACAAACC	GGACACCAGCAGGAGGAAG
CYP2E1-P8-Luc	TATGGGTTGGCAACATGTTCCT	GGACACCAGCAGGAGGAAG
CYP2E1-P9-Luc	GTGCTAGCAACCAGGGTGTTGA	GGACACCAGCAGGAGGAAG
CYP2E1-P10-Luc	CTGGGGGCCACCATTGCGGGAA	GGACACCAGCAGGAGGAAG

### Western blot analysis

Liver samples from HCC patients and control individuals were processed to protein extraction and analyzed by Western blot. Antibodies against HBx, CYP2E1 or HNF4α were incubated at 4°C overnight, followed by washing 5 min for 3 times with TBST (0.05% Tween-20 in Tris-buffered saline, TBS) and incubation with horseradish peroxidase conjugated secondary antibody (Zhongshan company Co., Beijing, China) for 2 h at room temperature. The membranes were washed again as described above, and the bands were detected by chemiluminescence for visualization. β-actin was used as an internal control. The expression level of CYP2E1 or HBx was represented by the optical density (OD) ratio to β-actin.

### RNA extraction, reverse transcription (RT) and PCR

Total RNA was isolated from cultured cells by TRIzol (Invitrogen, USA) according to the manufacturer’s instructions. Cells were lysed directly in the flasks, and RNA samples were stored at −70°C for later extraction. RNA concentrations were determined by absorbance at 260 nm, and the 260/280 nm absorption ratio of the samples was >1.9. The synthesis of cDNA and amplification of target gene was performed using a one-step RT-PCR kit (Takara, Dalian) using the specific primers listed in Table 2. The cDNA of GAPDH was adopted as an internal standard during RT-PCR analysis.

**Table 2:** RT-PCR and real-time PCR primers for CYP2E1, HBV RNA and GAPDH.

Genes	Sense (5′→3′)	Antisense (5′→3′)
CYP2E1	AATGGACCTACCTGGAAGGAC	CCTCTGGATCCGGCTCTCATT
HBV RNA	AGCAATGTCAACGACCGACC	GTGCGCAGACCAATTTATGCC
GAPDH	TCTGCTGATGCCCCCATGTTC	GGATGATGTTCTGGAGAGCCC

### Real time quantitative PCR

To quantitate the expression of CYP2E1 in human tissues or cultured cells, real time quantitative PCR was also performed. Preparation of RNA and synthesis of cDNA were carried out as described above. PCR amplication was performed with an Applied Biosystems PRISM 7500 Sequence Detector (Applied Biosystems, Foster City, CA, USA), using the Platinum SYBR Green qPCR SuperMix-UDG kit (Invitrogen, Carlsbad, CA, USA). The PCR procedure was heat at 94°C for 5 min followed by 40 cycles of 94°C for 30 s, 57°C 30 s and 72°C 45 s. The level of CYP2E1 expression was expressed as the ratio of CYP2E1 relative to GAPDH levels using the formula of 2^−ΔΔCt^.

### CYP2E1 promoter activity assay

HepG2 cells were plated into six-well plates at a density of 10^5 ^cells/well and grown overnight. The next day, cells were cotransfected with 1 µg of pCMV-2B–FLAG-X plasmid, 1 µg of CYP2E1 promoter luciferase reporter construct, and 1 µg of β-galactosidase reporter plasmid by the lipofectamine method (Invitrogen, USA) in the presence or absence of HBx expression vector. At 24 h post-transfection, cells were harvested for reporter gene assay. Activities of luciferase or β-galactosidase in the cell lysates were detected using the luciferase/β-galactosidase enzyme assay system (Promega). Luciferase activity was normalized to the β-galactosidase activity and calculated as an average of three independent experiments. To study the effect of HNF4α consensus site on CYP2E1 expression, mutations of this binding site on the CYP2E1 promoter were generated using the Quick Change Site-Directed Mutagenesis Kit (Stratagene, La Jolla, CA), and were named pGL3-mut-HNF4α. Transfection of mutant construct into HepG2 cells were performed as above and controlled to wild-type construct.

### HNF4α knockdown by RNA interference

Human HNF4α-specific siRNA (si- HNF4α): 5′-GGCAGUGCGUGGUGGACAAdTdT-3′ and 5′-UUGUCCACCACGCACUGCCdGdG-3′ and the scrambled control RNA (siNC): 5′-UUCUCCGAACGUGUCACGUdTdT-3′ and 5′-ACGUGACACGUUCGGAGAAdTdT-3′ was provided by GenePharma (Shanghai, China). HepG2 cells were transfected using Lipofectamine RNAiMAX (Invitrogen, USA) following the manufacturer’s instructions. After 24 h of transfection with siRNA, the samples were prepared for assays of RT-PCR and western blotting as indicated.

### E47 and C34 cell growth test

E47 cells, a human hepatoma cell line that constitutively expresses CYP2E1 (HepG2 cells transfected with plasmid pCI-neo containing CYP2E1 cDNA in the sense orientation), and C34 cells (HepG2 cells transfected with pCI-neo), which do not express CYP2E1 were used as an vitro model to test the effect of CYP2E1 expression level on hepatoma cell growth. E47 and C34 cells with the same density of 2×10^4^ cells/well were seeded in triplicate on 96-well plates and cultured for up to 6 days. Each day, the triplicate of cultured cells was harvested and subjected to CCK-8 assay at 450 nm. The amount of the formazan dye, generated by the activities of dehydrogenases in the HepG2 cells, is directly proportional to the number of living cells.

### Caspase-3 activity assay in C34 and E47 cells

Caspase-3 activities were determined in cell lysate by measuring proteolytic cleavage of the proluminescent substrates AC-DEVD-AMC (Calbiochem, La Jolla, CA). The fluorescence was detected to reflect the amount of released AMC (caspase-3, λex = 380, λem = 460). The results were expressed as arbitrary units of fluorescence (AUF) per milligram of lysate protein.

### In vivo mouse models and liver proteomics analysis

SV129 background CYP2E1 knockout mice were kindly provided by Dr. Frank J Gonzalez (Laboratory of Metabolism, National Cancer Institute, Bethesda, MD), and breeding colonies estabolished at Mount Sinai. SV129 wild type mice were purchased from Charles River Laboratory. Animal experiments were performed in Mount Sinai. Mice used in this study were all males at the age of 8 weeks, with body weight of 20–25 g, and fed with liquid dextrose diet (Bio-Serv, Frenchtown, NJ). When sacrificed, liver tissues were collected and rapidly excised into small fragments and washed with cold saline. With the use of the iTRAQ technique, a multifactorial comparative proteomic study between wild type and CYP2E1 knockout mice can be performed. MALDI plates were analyzed with a TOF/TOF 5800 mass spectrometer (AB Sciex). Functional annotation of protein was conducted using DAVID Bioinformatics Resources 6.7 (NIAID/NIH). For molecular pathway and network analysis of significantly changed proteins, the quantitative data were analyzed using Ingenuity Pathways Analysis (IPA).

### Statistical analysis

Data are presented as mean values ± SD. Differences between groups were analyzed by one-way ANOVA (SPSS 10.0 statistical software package, SPSS Inc., Chicago, IL) in all assays. P<0.05 was considered statistically significant.

## Results

### Enhanced HBx expression correlates with lowered CYP2E1 level in livers of HCC patients

**Figure 1:**
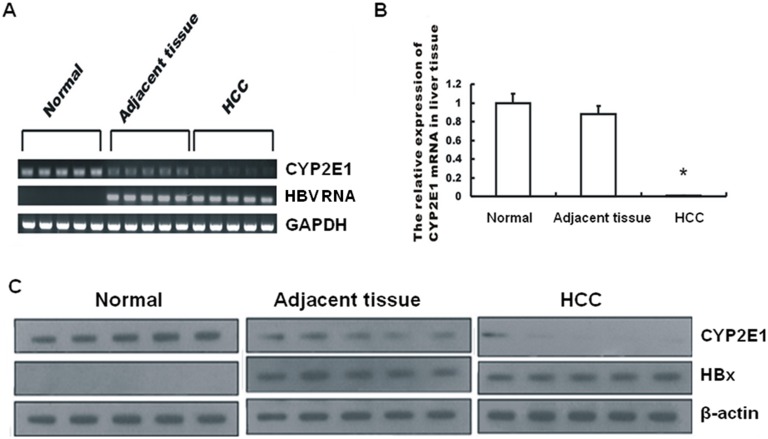
Enhanced HBx expression correlates with lowered CYP2E1 level in livers of HCC patients. (A) CYP2E1 and HBV RNA were determined by RT-PCR using GAPDH as an internal control in surgically resected human normal hepatic tissue, HCC tissue and its adjacent tissues. (B) CYP2E1 mRNA level was detected by real time quantitative PCR. (C) CYP2E1 and HBx protein were measured by Western blotting using β-actin as an internal control. The values were represented as the mean±standard deviation; *, P<0.001 vs. normal tissue or the adjacent tissue.

Since previous studies show that viral proteins play a critical role in HCC development, we analyzed the expression levels of HBV RNA and CYP2E1 in liver tissues from HCC patients. 5 pairs of HBV-positive primary HCC tissues, adjacent tissue of HCC and 5 cases of HBV-negative normal liver tissues were freshly resected from surgery. RT-PCR, real-time quantitative PCR, and western blot were performed to measure HBV RNA and CYP2E1 mRNA with a focus on correlation of HBx proteins levels and CYP2E1 amount. Results showed that expression of HBV RNA (Fig. 1A) and HBx protein (Fig. 1C) were higher in HBV-positive HCC livers. Conversely, CYP2E1 mRNA and protein levels were markedly lower compared to those of the normal livers (Fig. 1B and Fig. 1C). The inverse correlations between two proteins suggest that HBx may down-regulate CYP2E1 expression.

### Enforced expression of HBx decreases CYP2E1 gene expression in HepG2 cells

**Figure 2:**
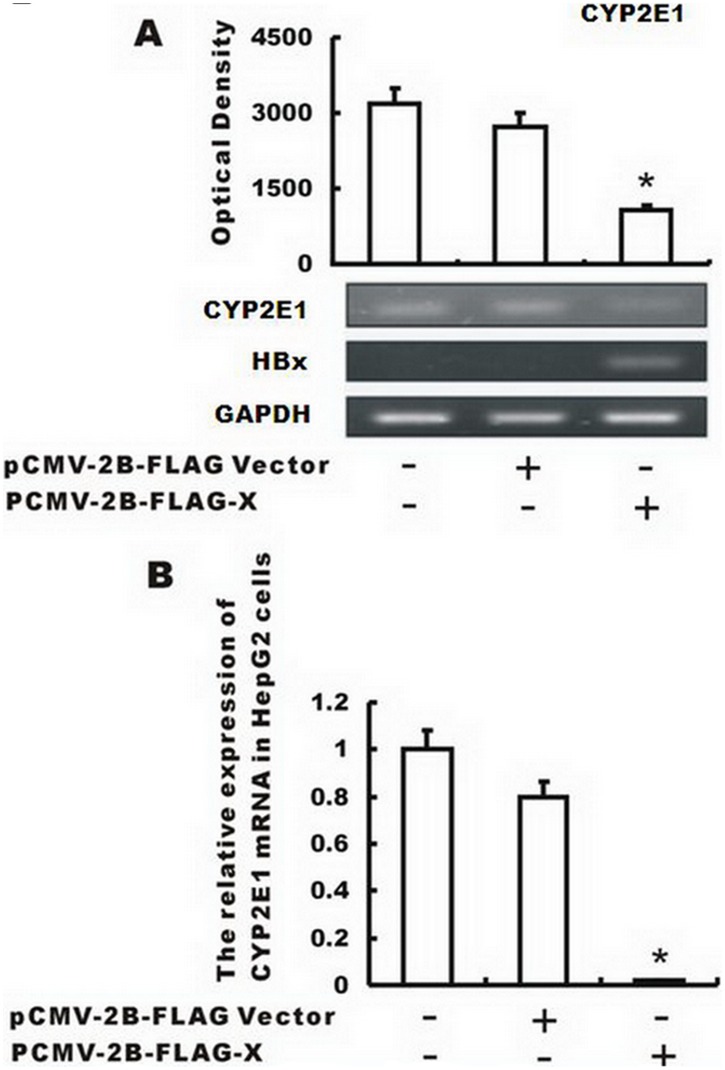
Enforced expression of HBx inhibits CYP2E1 gene expression in HepG2 cells. Plasmid expressing HBx (pCMV-2B–FLAG-X) or a control plasmid (pGL3 empty vector) was transfected into HepG2 cells. (A) Overexpression of HBx significantly inhibited CYP2E1 mRNA compared to the non-transfected cells or pGL3 empty vector transfected cells by RT-PCR with GAPDH as an internal control. (B) Effect of ectopic overexpression of HBx on CYP2E1 mRNA level was determined by real time quantitative PCR in HepG2 cells. The values were represented as the mean standard deviation; *, P<0.001 vs. the control group or the empty vector group.

To test whether HBx functions to inhibit CYP2E1 expression, a HBx-coding plasmid, pCMV-2B–FLAG-X, was introduced into HepG2 cells via transient transfection. Results showed that ectopic gene transfer increased mRNA of HBx in HepG2 cells (Fig. 2A). Overexpression of HBx significantly lowered CYP2E1 mRNA compared to the non-transfected cells or pGL3 empty vector transfected cells as shown by RT-PCR (P<0.001) (Fig. 2A) and real-time quantitative PCR (Fig. 2B). These results demonstrate that CYP2E1 gene expression can be negatively regulated by HBx at the transcription level.

### HNF4α plays a crucial role in controlling CYP2E1 expression

**Figure 3:**
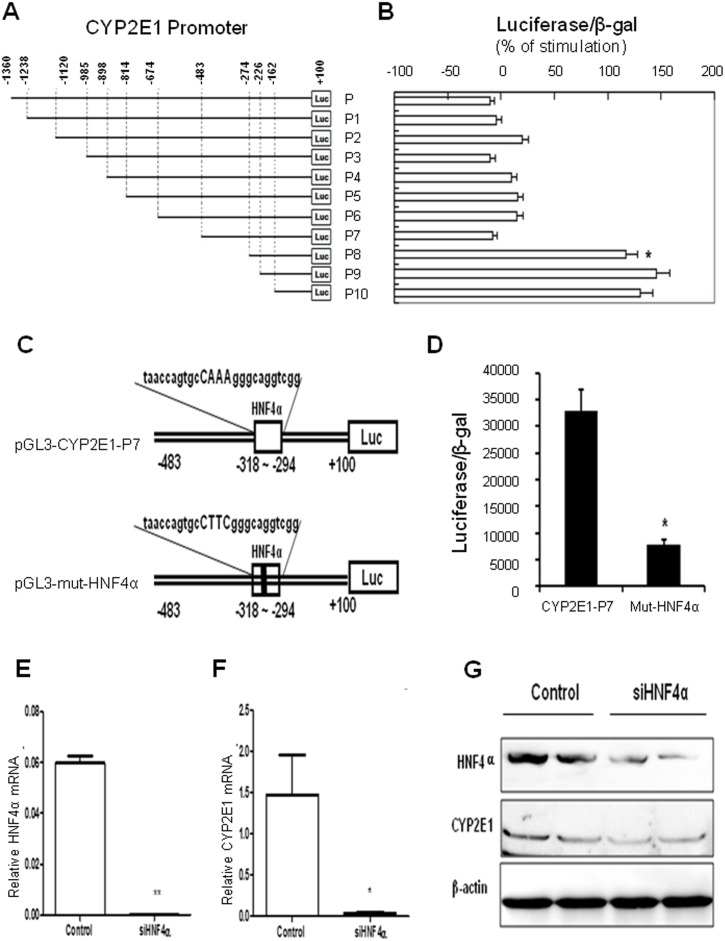
HNF-4α plays a critical role in CYP2E1 gene expression. (A) A schematic depiction of different human CYP2E1 promoter regions cloned into the pGL3-basic plasmid. The constructs were designated as CPY2E1-P-luc and its various deletion constructs CPY2E1-P1-luc to -P10. (B) Effects of HBx overexpression on promoter activities of different CYP2E1 promoter constructs and identification of a specific promoter region localized at −483∼−274 bp on the CYP2E1 gene 5′-flanking region upstream of the transcription start site which mediates the inhibition effect of HBx to repress CYP2E1 expression. HepG2 cells were cotransfected with one of the CYP2E1 promoter constructs and HBx expression plasmid or control plasmid. At 24 h post-transfection, cells were harvested for determination of relative luciferase activities. Results are expressed as percent of the corresponding control in the absence of HBx expression and represent mean±standard deviation. Results are expressed as percent of the corresponding untreated control cells and represent as mean±standard deviation; *, P<0.001 vs. construct CPY2E1-P7-luc. (C) Schematic representation of the consensus HNF4α binding element and its mutant forms on the human CYP2E1 gene promoter. Site-directed mutagenesis on HNF4α consensus binding was performed, and the mutant constructs were named pGL3-mut-HNF4α; (D) Effects of HNF4α binding site mutation on CYP2E1 promoter activity. (E–F) Effects of silencing HNF4α on endogenous HNF4α and CYP2E1 mRNA expression by real-time PCR, and (G) on their protein expressions by Western Blotting with β-action as internal control.

To molecularly define how HBx inhibits CYP2E1 expression, the human CYP2E1 promoter (−1360∼+100) was amplified and cloned onto the luciferase reporter plasmid pGL3 and named as pGL3-CYP2E1-P. Based on this plasmid, a series of pGL3 reporter plasmids harboring various lengths of the 5′-flanking region, spaced at 64–210 base pairs of the human CYP2E1 promoter were constructed and designated as pGL3-CYP2E1-P1 to –P10 (Fig. 3A). HepG2 cells were cotransfected with HBx expression vector and each of the reporter plasmids. As shown in Fig. 3B, HBx significantly inhibits CYP2E1 promoter activity in constructs pGL3-CYP2E1-P1 to –P7, but not pGL3-CYP2E1-P8 to –P10. These results indicate that the 5′-flanking region located at –483∼–274 base pairs upstream of the human CYP2E1 gene transcriptional start site is required for HBx to repress the human CYP2E1 promoter activity.

To precisely define the transcription factor mediating this inhibition effect, CYP2E1 promoter sequence was subjected to transcription factor binding sites search using an online software of MatInspector professional analysis (http://www.genomatix.de/en/index.html), and a consensus element for HNF4α was found at −318 to −294. As previous studies demonstrated that HNF4α regulates CYP2C9 and CYP2C19 gene expression [29], we tested if this HNF4α consensus site plays a role in CYP2E1 expression. Site-directed mutagenesis was subsequently performed on the construct pGL3-CYP2E1-p7 and the corresponding mutant was designated as pGL3-mut- HNF4α (Fig. 3C). Results showed that mutation of this site disrupted the basal CYP2E1 promoter activity by 77% (Fig. 3D), indicating that this DNA element is critical in controlling CYP2E1 transcription in HepG2 cells. To validate the role of HNF4α in the regulation of CYP2E1 expression, endogenous HNF4α expression was silenced with small interfering RNA (siRNA) in HepG2 cells, and the HNF4α knockdown effects was confirmed both on the mRNA and protein level (Fig. 3E and 3G). CYP2E1 mRNA and protein level markedly decreased when HNF4α was silenced (Fig. 3F and 3G). Together, these results showed that HNF4α plays a crucial role in controlling CYP2E1 expression in hepatocytes.

### Down-regulatory effect of HBx on CYP2E1 expression is mediated through HNF4α

**Figure 4:**
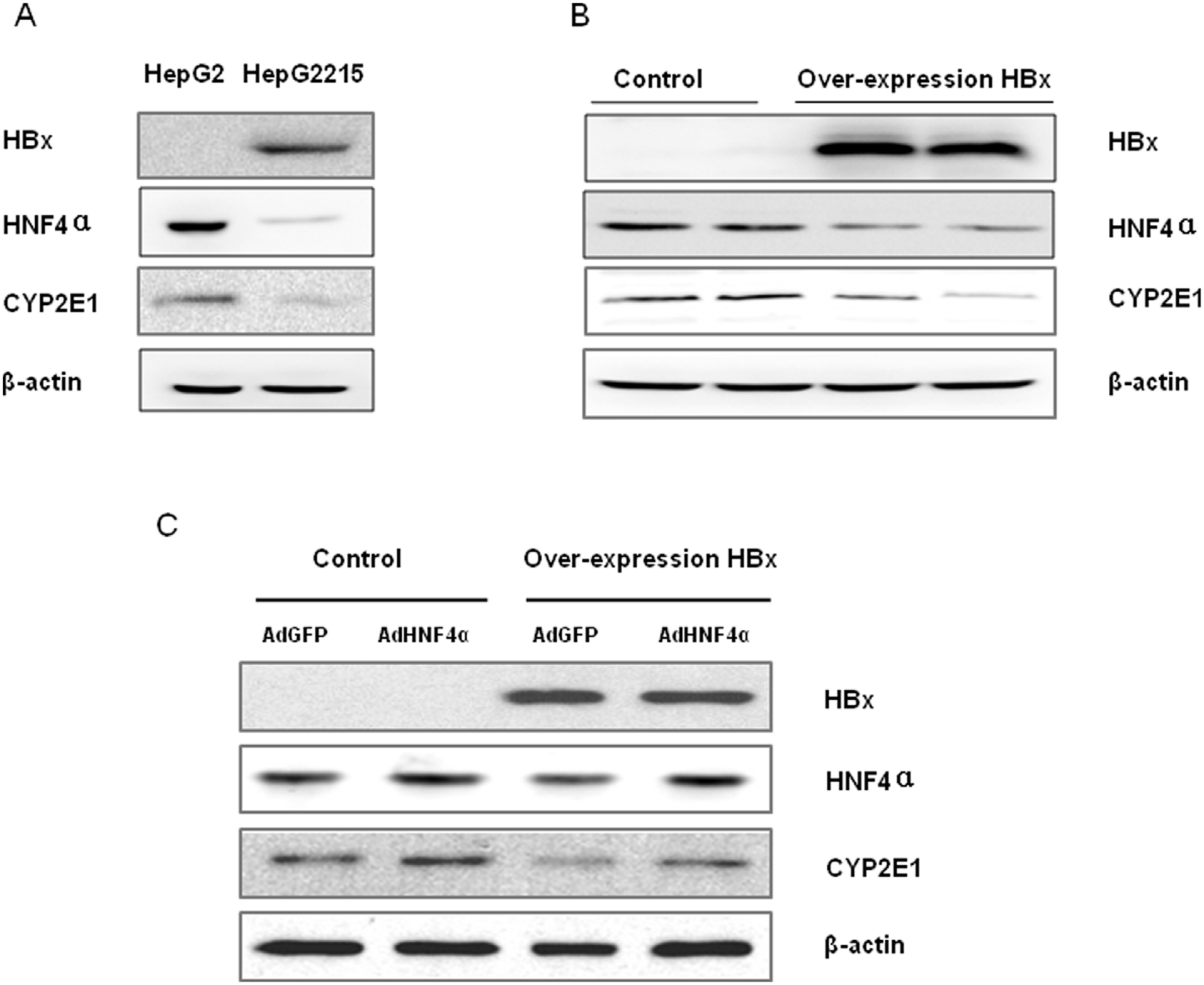
HBx inhibits CYP2E1 expression via downregulation of HNF4α. (A) HBV virus downregulated HNF4α and CYP2E1. HepG2215 cells, a cell line with the HBV genome integrated into the chromosome of HepG2 cells and capable of producing all HBV proteins, was used. Expression of HBx, CYP2E1 and HNF4α levels were measured with Western blotting. (B) Enforced expression of HBx protein downregulates CYP2E1 and HNF4α. HepG2 cells were transiently transfected with HBx-expression plasmid, and 24 hours post transfection, cells were harvested and cell lysate were used to determine HNF4α expression with Western Blotting. (C) HBx-induced CYP2E1 reduction could be rescued by HNF4α. HepG2 cells were transiently transfected with HBx-expression plasmid for 24 hours, then were infected with 1×10^9^ plaque-forming units of AdHNF4α, or the same amount of AdGFP as control for one more day, respectively, in HepG2 cells. Cells were harvested and protein expression of HBx, CYP2E1 and HNF4α levels were measured with Western blotting.

As previous studies from animal and clinical tissues showed that HNF4α levels were markedly decreased in HCC liver tissues [21], [22], we tested whether infection of HBV affects HNF4α levels in hepatocytes. For this purpose, we turned to HepG2.2.1.5, a cell line with HBV DNA sequences chromosomally integrated into HepG2 cells and capable of consistently expressing all the HBV encoded proteins. Western Blotting showed the stable overexpression of viral protein (HBx) in HepG2.2.15 was inversely correlated with significantly lower levels of HNF4α and CYP2E1 proteins compared to HepG2 cells (Fig. 4A). To confirm the role of viral protein HBx in regulating HNF4α expression, HBx-expression plasmid was transiently expressed into HepG2 cells. Western Blotting showed over-expression of HBx significantly inhibited HNF4α as well as CYP2E1 expression (Fig. 4B). To further validate that the down-regulatory effect of HBx on CYP2E1 gene expression was mediated through HNF-4α, we applied the adenovirus mediated overexpression of HNF-4α(AdHNF4α) with AdGFP as control in the presence or absence of HBx expression plasmid, respectively, in HepG2 cells. Western Blotting showed that CYP2E1 protein levels could be recovered, at least partially, by ectopic supplement of HNF-4α (Fig. 4C). Taken together, these results confirmed that HBx-induced CYP2E1 reduction could be rescued by ectopic supplement of HNF-4α protein expression.

### Expression of HNF4α and CYP2E1 inversely correlates with HBV infection in HCC liver tissues

**Figure 5:**
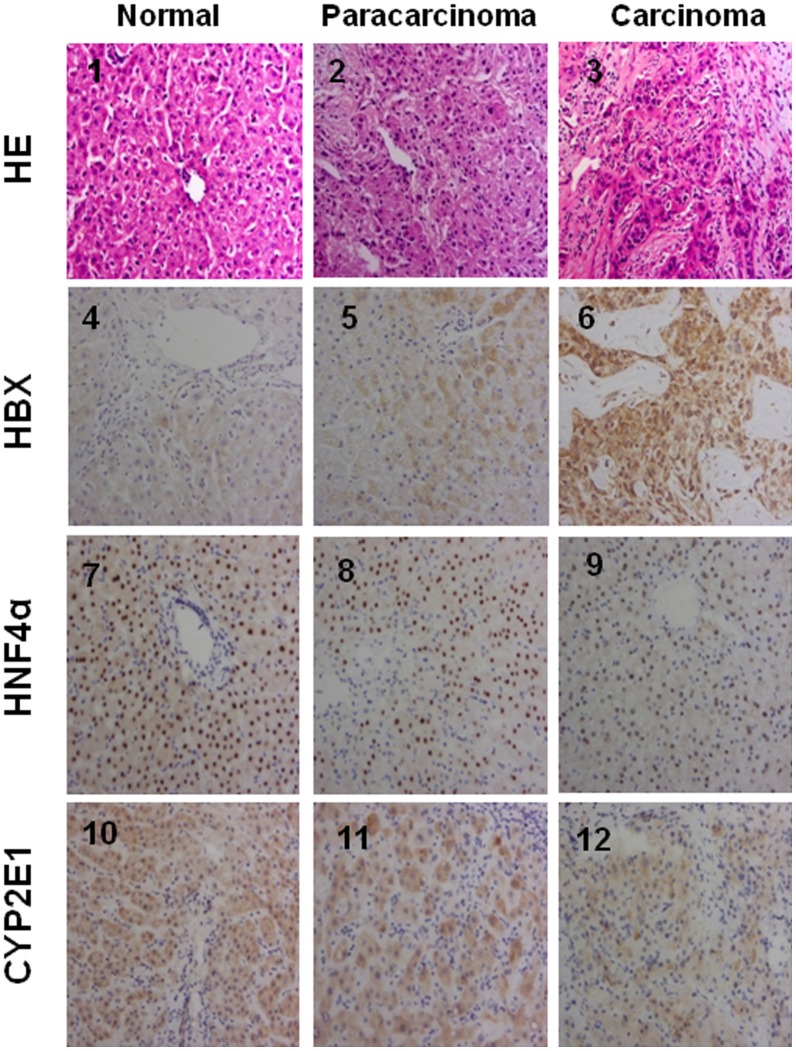
Immunohistochemical staining of HNF4α, CYP2E1 and HBx in human liver tissues. Row 1, morphology observation with HE staining. Row 2, HBx immunochemical staining. Row 3, HNF4α immunochemical staining. Row 4, CYP2E1 immunochemical staining. All HE staining or immunochemical staining were shown as typical fields from five cases of human normal, and five cases of paracarcinoma or HCC liver tissues.

To determine whether HNF4α and CYP2E1 are coordinately regulated in vivo, immunohistochemical (IHC) staining was performed in normal and HCC tissue. Morphological and IHC changes of liver tissues are shown in Fig. 5. HE showed the normal structure of liver tissue (panel 1), adjacent paracarcinoma tissue (panel 2) and typical carcinoma tissue (panel 3). HBx staining showed that normal liver tissue is absent of HBV infection (panel 4), whereas intensive HBx expression was observed in HBV positive HCC liver tissue (panel 6), and expression of HBx was much higher compared to the paracarcinoma area (panel 5). Conversely, HNF-4α levels were inversely correlated with HBx expression (panel 7 to panel 9) and positively correlated with CYP2E1 expression (panel 10 to panel 12). These results provide in vivo evidence for a synergistic change of HNF-4α and CYP2E1 expression under chronic HBV infection in human livers.

### Increased CYP2E1 inhibits HepG2 cell growth

**Figure 6:**
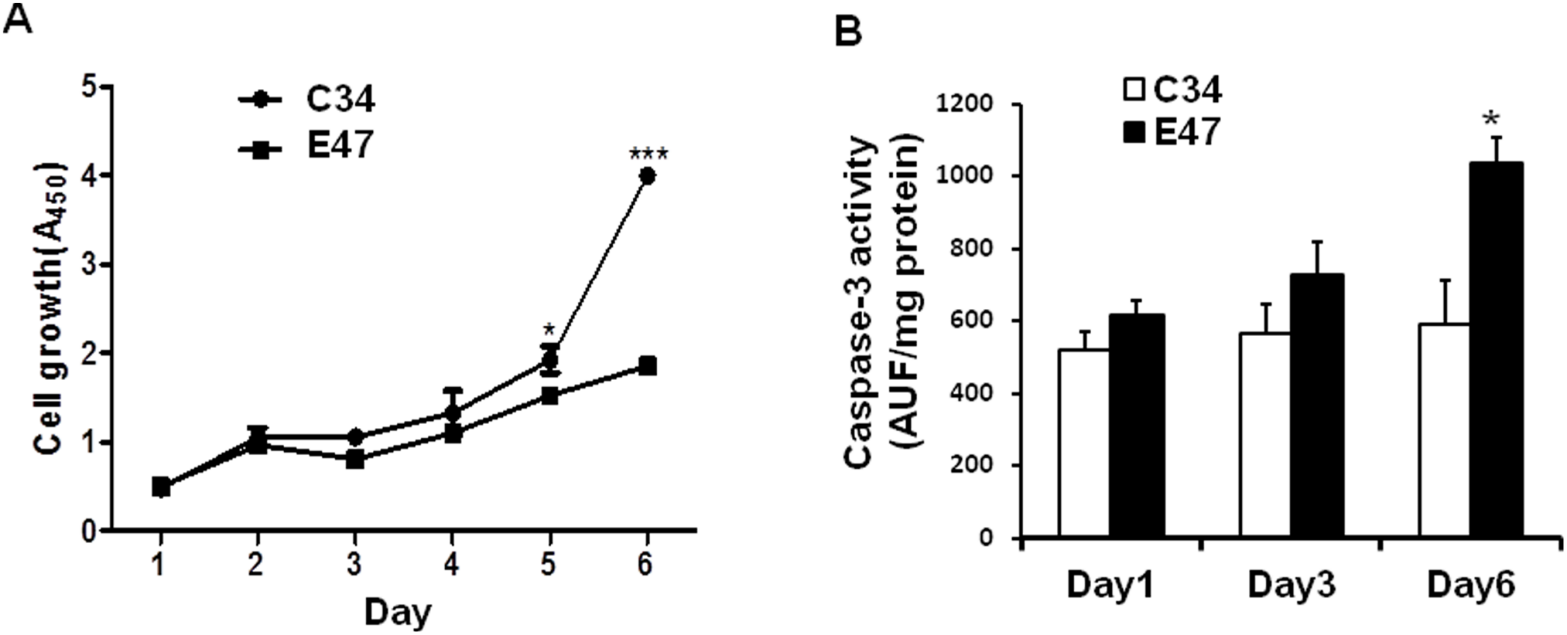
Cell proliferation and caspase-3 activity assay. (A) Stable overexpression of CYP2E1 inhibits the cell growth of HepG2 cells. E47 and C34 cells, two stably transfected HepG2 cell lines harboring CYP2E1 recombinant gene or its empty vector respectively, were seeded with the same number in triplicate on 96-well plates and cultured for up to 6 days. Each day, the triplicate of cultured cells was harvested and subjected to CCK-8 assay at 450 nm. The amount of the formazan dye, generated by the activities of dehydrogenases in cells, is directly proportional to the number of living cells. (B) Activities of caspase-3 increased in HepG2 cells which stably expresses ectopic CYP2E1. E47 and C34 cells were seeded in triplicate on 96-well plates. Cells were harvested on day 1, 3 and 6, and cell lysates were used to test the activities of caspase-3 by measuring proteolytic cleavage of the proluminescent substrates AC-DEVD-AMC. The fluorescence was detected to reflect the amount of released AMC (caspase-3, λex = 380, λem = 460). The results were expressed as arbitrary units of fluorescence (AUF) per milligram of lysate protein.

In order to understand the consequences of decreased CYP2E1 in hepatocarcinogenesis, we tested the effect of CYP2E1 levels on hepatoma cell growth. E47 cells, a stably transfected HepG2 cell line with CYP2E1 overexpression was compared with C34 cells harboring only empty vector in the same host cell as the control. Cells were loaded with the same amount in 96 wells and were cultured up to 6 days. Cell growth rate was determined each day with a CCK8 kit. Result showed that E47 cells demonstrated significantly lower growth rate compared to C34 cells (Fig. 6A), indicating that the decreased expression of CYP2E1 promotes hepatoma cell growth. As a strong generator of reactive oxygen species (ROS), elevated CYP2E1 expression participates in promoting cell apoptosis [34], [35], we tested whether this CYP2E1-induced reduction in cell growth was due to enhanced cell apoptosis. Activities of caspase-3 were determined in C34 and E47 cells post-seeding onto 96-well plates on day 1, 3 and 6. Results showed that activities of caspase-3 were increased in E47 cells compared to C34 cells and the difference was statistically significant on day 6 (Fig. 6B), indicating that decreased cell growth in E47 cells may be attributed, at least in part, to enhanced cell apoptosis.

### Impacts of CYP2E1 absence on multiple cellular pathways

To better understand the effects of decreased CYP2E1 on hepatocyte functions at the molecular level, liver tissues from adult CYP2E1-knockout mice were collected and subjected to proteomics analysis to be compared with wild type mice as the control. Principal component analysis (PCA) was conducted with Matlab statistical software. All identified proteins with quantitative data were tested with p<0.05. The quantitative mass spectrometry data from CYP2E1 knockout mice which was compared to control showed that, 23 proteins were significantly increased with the fold >1.5. Among the up-regulated proteins, 5 are involved in protein synthesis which includes small and large ribosomal proteins as well as initiation factor with 2 of those demonstrating the highest fold change (Table 3). Proteins which are involved in amino acid or carbohydrate catabolism, electron transfer and ATP synthase were also increased, all of which may act to supply increased energy production for protein synthesis. In addition, some of the increased proteins were also those participating in other aspects of liver functions, such as biotransformation or xenobiotics metabolism, serum protein for transportation, and histone as a chromosome constituent. In particular, some of the increased proteins such as keratins and cdc42 functions to maintain epithelial hepatocyte structure or cell cycle control. Enhanced levels of these proteins were previously shown in HCC [30], [31].

**Table 3:** Proteomic results: increased.

Accession	Gene Symbol	Protein description	KO/WT
**Protein synthesis**			
P60843	Eif4a1	Eukaryotic initiation factor 4A–I	99.083
P62849	Rps24	40 S ribosomal protein S24	36.644
P62908	Rps3	40 S ribosomal protein S3	9.036
P14148	Rpl7	60 S ribosomal protein L7	5.495
P47963	Rpl13	60 S ribosomal protein L13	1.770
**Protein/amino acid catabolism**			
P46471	Psmc2	26 S protease regulatory subunit 7	83.176
Q9QXF8	Gnmt	Glycine N-methyltransferase	4.285
Q8QZR5	Gpt	Alanine amino transferase 1	1.820
**Carbohydrate catabolism**			
A2AJL3	Fggy	FGGY carbohydrate kinase domain containing protein	84.723
Q91×44	Gckr	Glucokinase regulatory protein	1.977
**Electron transfer/energy production**			
P03930	Mtatp8	ATP synthase protein 8	9.462
Q9D0M3	Cyc1	Cytochrome c1	6.668
**Biotransformation/xenobiotics metabolism**			
P24456	Cyp2d10	Cytochrome P450 2D10	11.803
P37040	Por	NADPH–cytochrome P450 reductase	7.447
Q9DBG1	Cyp27a1	Sterol 26-hydroxylase	4.656
Q9DCM2	Gstk1	Glutathione S-transferase kappa 1	3.467
**Hepatocyte structure maintenance**			
P11679	Krt8	Keratin, type II cytoskeletal 8	12.474
P05784	Krt18	Keratin	5.649
**Transport/signaling/chromosome**			
Q00724	Rbp4	Retinol-binding protein 4	84.723
P29391	Ftl1	Ferritin light chain 1	9.727
P60766	Cdc42	Cell division control protein 42 homolog	2.938
Q64374	Rgn	Regucalcin	1.542
P62806	Hist1h4a	Histone H4	4.699

The proteins whose levels are increased in CYP2E1 knockout versus wild type (WT) mice and directly related to carbohydrate, protein or amino acid catabolism, or biotransformation are listed. The relative protein expression level in knockout versus wild-type mice (KO/WT) is shown.

On the other hand, 18 proteins were shown to be significantly decreased with the fold<0.5 (Table 4). Among the down-regulated proteins, 2 of the most significantly decreased proteins are CYP2E1 and aldehyde dehydrogenase, both of which were known to be critical players in ethanol metabolism [10]. Notably, among the significantly decreased are 4 proteins encoded by Ogdh, Hadha, Acadm, Hmgcs2, which participating in metabolism of long-chain or medium-chain fatty acids, krebs cycle or ketogenesis. In particular, all of these are mitochondria proteins involved in lipid metabolism. There are also decreases in proteins with functions such as glucose or amino acid metabolism, serum protein and DNA binding proteins.

**Table 4:** Proteomic results: decreased.

Accession	Gene Symbol	Protein description	KO/WT
**Biotransformation/xenobiotics metabolism**			
Q05421	Cyp2e1	Cytochrome P450 2E1	0.047
Q9CPU0	Glo1	Lactoylglutathione lyase	0.061
P47738	Aldh2	Aldehyde dehydrogenase	0.394
**Fatty acid catabolism/ketogenesis**			
Q60597	Ogdh	2-oxoglutarate dehydrogenase	0.437
Q8BMS1	Hadha	Trifunctional enzyme subunit alpha	0.258
P45952	Acadm	Medium-chain specific acyl-CoA dehydrogenase	0.251
P54869	Hmgcs2	Hydroxymethylglutaryl-CoA synthase	0.101
**Glucose/amino acid metabolism**			
Q05920	Pc	Pyruvate carboxylase	0.331
O35490	Bhmt	Betaine–homocysteine S-methyltransferase 1	0.078
O08749	Dld	Dihydrolipoyl dehydrogenase	0.011
P97328	Khk	Ketohexokinase	0.492
**Transport/cell motility/chromosome**			
Q921I1	Tf	Serotransferrin	0.340
Q60597	Ogdh	2-oxoglutarate dehydrogenase	0.437
P07724	Alb	Serum albumin	0.449
Q8VDD5	Myh9	Myosin-9	0.125
RRsp|A	Ttn	REVERSED Titin	0.011

The proteins whose levels are decreased in CYP2E1 knockout versus wild type (WT) mice and directly related to carbohydrate, lipids, protein or amino acid catabolism, or biotransformation are listed. The relative protein expression level in knockout versus wild-type mice (KO/WT) is shown.

## Discussion

It is well known that CYP2E1 is a highly inducible enzyme whose expression changes under various circumstances. For example, CYP2E1 is induced by acute and chronic alcohol consumption resulting in the enhancement of their metabolism and also induced by ketone bodies such as acetone in diabetic patients [11]. During the biotransformation of exogenous and endogenous compounds by this enzyme, various reactive oxygen species (ROS) are produced which could lead to tissue damages [32], [33]. As CYP2E1 is a potent generator of ROS, the finding that CYP2E1 actually decreased in HCC is rather intriguing and may raise a number of questions. First, what is the function of decreased CYP2E1 in HCC development? Over expression of CYP2E1 in HepG2 cells and CYP2E1 knockin mice showed increased oxidative stress and cytotoxicity to the cell and liver injury [33], and stable transfection of CYP2E1 in E47 cells showed increased apoptosis compared to C34 cells upon ethanol treatment [34], [35]. In addition, other pathological changes, such increased ethanol-toxicity and accumulation of fat, were also documented in CYP2E1 knockin mice but not in CYP2E1 knockout mice [36]. Therefore it is reasonable to speculate that decreased level of CYP2E1 may cause lower cytotoxicity and apoptotic rate, thus favoring tumor growth. Indeed, decreased CYP2E1 has been previously shown in rat and human HCC tissues, and associated with poor prognosis of HCC [15]–[17]. In this study, we have shown that CYP2E1 was decreased in HCC tissues, thus adding further proof for downregulation of CYP2E1 in HCC. We have shown E47 cells with CYP2E1 expression have a significantly lower growth rate compared to C34 cells without ectopic CYP2E1 gene (Figure 6), suggesting that decreased CYP2E1 promotes hepatoma cells growth. With proteomics analysis, we have identified significantly differently expressed liver proteins that have not been previously reported in CYP2E1 knockout mice which may play a role in promoting hepatocarcinogenesis. For example, lack of CYP2E1 in HCC may decrease hepatic metabolism for xenobiotics including ethanol and procarcinogens, and a prolonged effect of this change may predispose liver to enhanced cytotoxicity. Also, decreased lipid metabolism (as shown in Table 4) may exacerbate liver steatosis and oxidative stress. Meanwhile, enhanced protein synthesis as evident by significant increases of multiple ribosomal proteins and initiation factors (see Table 3) may promote cell growth. Our study thus broadens the current understanding for the role of CYP2E1 in HCC development. The impacts of all these changes associated with lack of CYP2E1 on HCC development are currently unclear and merits further investigation.

How is CYP2E1 downregulated in HCC? It is known that CYP2E1 has been a focus in alcoholic liver diseases which has been intensively investigated in the past two decades. Regulation of this enzyme occurs at transcriptional, post-transcriptional and post-translational levels [12]. Multiple transcriptional factors have been identified to activate the CYP2E1 promoter, such as HNF-1α in rat [37], Sp1 and NF-κB in rabbit [38], and STAT5, STAT6, NFATc1 [39], [40] and GATA4 and NR5A2 (fetoprotein transcription factor) in human [41]. In adult animals, CYP2E1 is post-transcriptionally regulated through mRNA and protein stabilization [39]. In this study, we have identified HNF-4α as a critical activator controlling CYP2E1 expression. Supported for this concept are: (1) a consensus HNF-4α binding site was found at −318 to −294 bp on the CYP2E1 promoter region; (2) mutation of this site dramatically disrupted CYP2E1 promoter activity; (3) silencing of endogenous HNF-4α in HepG2 cells significantly decreased CYP2E1 expression both at mRNA and protein levels. To the best of our knowledge, this is the first evidence showing that HNF-4α plays a critical role in regulation of CYP2E1 expression. HNF-4 α is one of the critical hepatocyte enriched nuclear factors in the maintenance of liver architecture and function, and down-regulation of which has been repeatedly shown in rodent and human HCC [21], [22]. Importantly, forced expression of HNF-4α dramatically inhibits the epithelial mesenchymal transition (EMT) of hepatocyte, generation of the cancer stem/progenitor cells [23] and proliferation of HCC cells, thus preventing hepatocarcinogenesis in rats [23], [24]. Therefore, identification of CYP2E1 as a new target of HNF-4α expands our understanding for its function in HCC.

What provokes HNF-4α downregulation in HCC? As the majority of chronic hepatitis and cirrhosis patients are at high risk of liver cancer, substantial evidence has revealed that HBV proteins, in particular HBx, play an important role in the development of HCC. Several studies showed that HNF4α promotes HBV replication by binding to the core promoter of virus genes [25]. On the other hand, expression of HNF4α differs with different outcome of HBV infection, being significantly higher in patients with severe hepatitis B(SHB) than those with chronic hepatitis B(CHB) and liver cirrhosis(LC) [26], two well-established risks factors for CHB-associated hepatocarcinogenesis. In the current study, we demonstrated that HNF-4α was decreased by enforced expression of HBx in cultured HepG2 cells, and that the level of HNF-4α was significantly lower in HepG2.2.15 cell line and in HBV positive human HCC liver tissues. Our results thus identify HNF-4α as a mediator for chronic HBV infection-associated CYP2E1 down-regulation in promoting HCC development.

As chronic HBV infection is also associated with hepatic inflammation, and there is decreased expression of CYP2E1 by pro-inflammatory cytokines, such as IL-1β, IL-6 and TNF-α [42], [43], the possibility of its downregulation by HBV-induced immune responses cannot be excluded. In addition, HBV is known to encode four major proteins, HBs, HBc, HBp and HBx. HBs has also been suggested to play a pathogenic role in HCC [44]. Whether HNF-4α and CYP2E1 can be downregulated by HBs is yet to be determined. The increased CYP2E1 levels in para-cancerous HCC tissue have been suggested to promote tissue damage and cancerous transformation possibly due to increased production of ROS [17]. Therefore, functions of CYP2E1 at different stage in the development of HCC merits more detailed investigation.

In addition, previous study in liver-specific HNF4α knockout mouse model proved that HNF4α plays a critical role in controlling a subset of sexually dimorphic P450 gene expression via the altered expression of liver transcription factors both positively for HNF1α, C/EBPα, and C/EBPβ and negatively for HNF3α, HNF3β, HNF6 and the HNF4α coactivator PGC-1α [45]. Besides, androgen pathway was demonstrated in vitro and in vivo to increase HBV transcription, including HBx mRNA synthesis and subsequent protein expression [46]–[48]. Therefore, the findings of HBx/HNF4α/CYP2E1 pathway proposed in this study may be differently modulated by sex hormones. Future studies of the molecular mechanism(s) for HNF4α and other liver factors in regulation of CYP2E1 expression in condition of HBV infection should include both genders, which may help to better understand the gender disparity in HBV-related HCC.

In conclusion, our results demonstrate that CYP2E1 is controlled by HNF-4α, and both of these proteins are down-regulated by HBx. Elucidation of the novel pathway of HBx/HNF-4α/CYP2E1 might provide insight into molecular mechanisms for hepatocarcinogenesis, particularly, under chronic HBV infection.
